# Anti-Glycan Autoantibodies Induced by *Helicobacter pylori* as a Potential Risk Factor for Myocardial Infarction

**DOI:** 10.3389/fimmu.2020.00597

**Published:** 2020-04-08

**Authors:** Riccardo Negrini, Vincenzo Villanacci, Claudio Poiesi, Antonella Savio

**Affiliations:** ^1^Department of Laboratory Medicine, Presidio di Gardone VT-ASST Spedali Civili, Brescia, Italy; ^2^Institute of Pathology, ASST Spedali Civili, Brescia, Italy; ^3^Institute of Microbiology and Virology, ASST Spedali Civili, Brescia, Italy; ^4^Histopathology and Cytology Department, The Royal Marsden NHS Foundation Trust, London, United Kingdom

**Keywords:** myocardial infarction, *Helicobacter pylori*, molecular mimicry, autoimmunity, histo-blood group antigens, thrombophilia

## Abstract

A number of epidemiological studies have evaluated the potential association between *H. pylori* and cardiovascular disease, but with contrasting results. We have previously shown that *Helicobacter pylori* infection is able to induce in mice and humans autoantibodies cross-reacting with histo–blood group Lewis antigens, expressed in different organs and in plasma glycoproteins and glycolipids. The aim of this study was to assess whether immunization of animals with *H. pylori* might induce myocardial histopathological changes. We have retrospectively examined, in detail, the histology of archived organs from mice and rabbits immunized with *H. pylori* in our previous studies. Human sera and cross-reacting monoclonal antibodies were also tested against bacterial preparations and tissue sections. Areas of myocardial necrosis, associated with coronary thrombotic occlusion, were found in 5 of 20 mice and 2 of 5 rabbits previously immunized with suspensions of *H. pylori*. No similar lesions were found in control animals, suggesting a causal link with *H. pylori* immunization. The animals bearing myocardial lesions had not been infected but only immunized months earlier with parenteral injections of dead *H. pylori* cells. This strongly suggests that immunization, by itself, might play a causative role. We propose that the cross-reactive autoimmune response induced by *H. pylori* could promote thrombotic occlusion through direct endothelial damage or by perturbing the coagulation process.

## Introduction

Myocardial infarction is one of the main causes of mortality worldwide. In the great majority of cases it is caused by a sudden occlusion of a coronary artery, on the background of atherosclerosis. A number of immunological and experimental studies have demonstrated the importance of inflammation in the initiation and progression of atherosclerosis, including the final thrombotic occlusion (1, 2). Considerable evidence has emerged that this inflammatory process is sustained, at least in part, by bacteria or viruses, particularly *Chlamydia pneumoniae* (3), *H. pylori* (4, 5), and *Cytomegalovirus* (6). It has been proposed that the effect of infection on the vascular wall may be direct, or indirect, by inducing an autoimmune inflammatory response (7).

In 1988 we generated the first monoclonal antibodies, in mice, against *H. pylori*, in order to use them for the immunohistochemical detection of this bacterium in human gastric biopsy sections. We observed that immunizing mice with *H. pylori* induced a marked immune reaction against gastric mucosal mucins (8). These antibodies were easily detected using a simple indirect immunoperoxidase or immunofluorescence test, even with highly diluted sera. In detail, more than 30% of the mouse monoclonal antibodies screened as specific for *H. pylori* in relation to other bacteria, strongly cross-reacted with human gastric mucins. These mouse antibodies also cross-reacted with the gastric epithelium of mice of the same species the antibodies were produced from (9). In other words, these monoclonal antibodies, as well as the antibodies present in sera from these animals, represented true autoantibodies.

Subsequently we have shown that an identical autoimmune response takes place in humans. In fact, most patients with *H. pylori* infection produce autoantibodies cross-reacting with autologous gastric mucins, whose presence is also correlated with the degree of gastric mucosal inflammation and atrophic changes (10).

The phenomenon of cross-reactivity relies on molecular mimicry, where an antigen of a micro-organism shares structural similarities with the host's molecules. This can induce the breakdown of the self-tolerance and a consequent cross reacting autoimmune response, as firstly exemplified by rheumatic fever (11).

The analysis of our monoclonal antibodies revealed that the cross-reaction involved histo-blood group like carbohydrate antigens (Lewis X, Lewis Y, H type 1), expressed by the lipopolysaccharide (LPS) of *H. pylori* (12).

**Figure 1:**
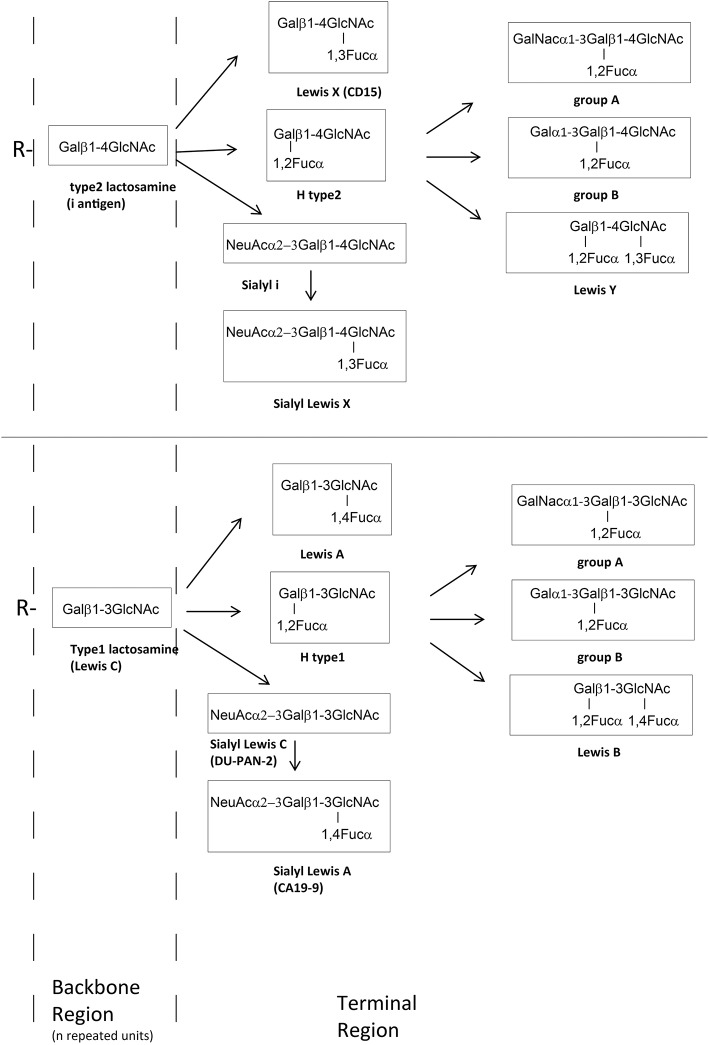
Schematic glycosylation pathway of the most important external glycan structure present on glycoproteins (N- and O- glycans) and glycolipids. The backbone chains (type 1 or 2 chain) are terminated by fucose (Fuc), galactose (Gal), GalNAc, or sialic acid (NeuAc) forming histo–blood group antigens antigens such as A, B, O, or Lewis type antigens.

Lewis X (LeX) and Lewis Y (LeY) are expressed by most *H. pylori* strains, while H type 1 is present in a minority of strains (up to 20%) (13). In humans, ABH and Lewis blood group antigens are usually expressed at the ends of long polylactosamine (Gal-GlcNAc) scaffolds bound to glycolipids or glycoproteins (Figure 1) (14). These carbohydrate antigens are synthesized by specific glycosyltransferases encoded by the *ABO, FUT1, FUT2*, and *FUT3* genes. These enzymes add monosaccharides to a precursor molecule sequentially, creating new antigenic epitopes (15). In erythrocytes, platelets and endothelial cells, the blood group related antigens are mainly mounted on type 2 polylactosamine chains (Figure 1), with repeating Galβ1 → 4GlcNAc disaccharide units, whereas, in the epithelium of the gastrointestinal, respiratory, genitourinary tracts, in exocrine secretions and in plasma, they are mainly mounted on type 1 chains (Galβ1 → 3GlcNAc).

Similarly to other gram negative bacteria, the outermost portion of the *H. pylori* LPS molecule is represented by a highly immunogenic O-antigen polysaccharide chain. A typical *H. pylori* LPS O-antigen consists of partially fucosylated, glucosylated, or galactosylated polylactosamine backbone chains with a significant degree of inter- and intra-strain heterogeneity as shown by their reactivity with different monoclonal antibodies (12, 16, 17).

After our initial reports on antigenic mimicry of *H. pylori*, a considerable number of studies have followed and papers are being published on the possible role of *H. pylori* in different extra-gastric diseases, via proven or suspected autoimmune pathogenesis (18). The beneficial effect of eradicating *H. pylori* infection has been demonstrated in some of these diseases (19). In thrombocytopenic purpura, e.g., the international consensus guidelines recommend *H. pylori* testing (20).

Particularly important is the connection with coronary heart disease (CHD). Although the published data so far are not entirely concordant, recent analysis of previous studies have provided convincing evidence of a possible role of *H. pylori* (21, 22). Recent studies have also demonstrated a trend toward a decrease in the occurrence of CHD after *H. pylori* eradication (23) and association between *H. pylori* infection with subclinical significant coronary artery stenosis in a healthy population (5).

Ever since we observed that the autoimmune reaction induced by *H. pylori* involved not only the stomach, but also, to a lesser extent, epithelia of other extra-gastric tissues (9), we have stored organs of many animals in formalin or paraffin-embedded blocks for retrospective study.

Now we have evaluated, in depth, the archived hearts from mice and rabbits immunized by subcutaneous injection of *H. pylori* during our previous studies, histologically. Unequivocal histological features of infarction were observed in some of these hearts in serial full thickness H-E stained sections. To gain further insight into significance of this finding, we extended our research using archived sera, gastric mucosal biopsies and bacterial preparations from a panel of patients who had undergone gastroscopy during our previous studies. The immunological reactions of anti *H. pylori* monoclonal antibodies with bacterial preparations and tissue sections were also examined.

## Materials and Methods

### Bacteria

Immunization of mice and rabbits with *H. pylori* was performed using 12 clinical isolates subcultured on 6% horse blood agar at 37°C in a microaerobic atmosphere (24). Bacteria were harvested and washed in phosphate-buffered saline (PBS), pooled together in approximately equal proportion to give a final concentration of 10^9^ cells/ml. The suspension was divided into 1 ml aliquots and frozen at −80°C for later use. Immunization with *Campylobacter jejuni* (*C. jejuni*) was performed using a pool of 5 clinical isolates following the procedure described for *H. pylori*. Immunization with a pool of gram negative bacteria was performed with three clinical isolates of *E. coli, K. pneumoniae*, and *P. mirabilis*.

The two *H. pylori* strains derived from two patients with a previous history of myocardial infarction, as described below, were re-grown from liquid nitrogen. These strains were not among the 12 *H. pylori* isolates used for mouse and rabbit immunization.

The control isolates P466 and MO19 are two *H. pylori* strains whose O-antigen region is well recognized. The P466 LPS expresses both LeX and LeY epitopes (Lipid A-core-LeX polymer plus an LeY terminus). The O-antigen region in the MO19 LPS expresses LeY in the monomeric form (Lipid A-core-LeY) (17).

### Mice and Rabbits

This study was performed on 4-μm-thick sections cut from formalin-fixed, paraffin-embedded organs stored for years at room temperature or from organs stored in vials of 5% formalin and only recently processed and embedded in paraffin. These organs were from mice and rabbits used in our previous experimental studies (8–10, 24). H-E stained serial sections were obtained at 25-μm intervals.

#### Mice

Test and control mice belonged to a unique lot of 72 Balb/c mice used in two different projects aimed at producing monoclonal antibodies for diagnostic purpose: antibodies against *H. pylori* (n°38) and antibodies against *C. jejuni* (n°24). The control group comprised 34 mice: the 24 mice immunized with *C. jejuni*, 2 mice immunized with a pool of gram negative bacteria, and 8 mice not immunized.

Immunization was performed by subcutaneous injections of 1 ml aliquot of bacterial suspensions (10^9^ cells in sterile PBS only, as described above—no adjuvant). A total of 3–5 injections were given at 2-weeks intervals. The injections did not cause adverse reactions nor discomfort or stress. The animals were housed in cages under temperature 25 ± 2°C and 12 h dark-light cycle and were fed with standard diet and watered *ad libitum*. Except for a few mice killed by decapitation to obtain spleen lymphocyte-myeloma cell fusions, mice were kept in cages without any further stress up to a maximum of ~1 year after the end of the immunization cycle.

#### Rabbits

The organs were from 11 New Zealand rabbits of whom 8 had been immunized for the production of polyclonal antisera, five with *H. pylori*, and three with *C. jejuni*, and three had not been immunized.

Immunization was performed as described for the mice but with a total of 5–10 injections at 4–8 weeks intervals. Rabbits were individually housed in cages under temperature 21 ± 2°C and 12 h dark-light cycle and were fed with standard diet and watered *ad libitum*. Blood samples (5–10 mL) were collected via the marginal ear vein 15–20 days after each booster injection over a period of 12–15 months. At the end of the immunization cycle, the rabbits were kept in cages without any further stress up a maximum period of 2 years.

All animals were monitored daily by trained animal technicians.

### Patient Sera

Aliquots of anonymized residual serum specimens stored at −80°C from two groups of patients, who had undergone to gastroscopy during our previous studies, were used (9, 10, 24). Amongst these patients there were two males aged 39 and 61 with a history of myocardial infarction, previously identified by cross-referencing cardiac and endoscopy databases. Blood samples from healthy anonymous donors were provided from our hospital transfusion service.

### Monoclonal Antibodies

Antibodies CB-10 (IgM), specific for LeX, CB-4 (IgG1), 1E52 (IgM), and HpN35 (IgM), specific for LeY, 4D2 (IgM), specific for H-type1 and A105 (IgM), specific for LeB, were obtained by immunizing Balb/c mice with a pool of *H. pylori* strains, as previously described (8, 24). Antibody KM93 (IgM), specific for sialyl-LeX was from Calbiochem (San Diego, CA). Antibody 19-OLE (IgM), recognizing H type 2 antigen was from Invitrogen.

### Synthetic Antigens Used to Define Monoclonal Antibody Specificities

The human serum albumin (HSA)- and bovine serum albumin (BSA)-conjugated carbohydrates were obtained from Isosep AB (Uppsala, Sweden). The antigens were provided with an average of 17 and 20 carbohydrate moieties per protein.

Glycoconjugates were diluted in pH 9.6 carbonate/bicarbonate buffer at a concentration of 1 μg/well and coated overnight onto polystyrene microwells at 4°C. The wells were washed, overcoated with 5% normal rabbit serum for 60 min and then incubated 60 min. with each monoclonal antibody (5 μg/ml in PBS-1% BSA). Peroxidase-labeled rabbit anti-mouse immunoglobulin was used as the second antibody. After washing, the reactions were developed with TMB substrate and read at 450 nm.

### Immunohistochemical Staining

The demonstration of autoantibodies in sera was performed using a simple indirect immunoperoxidase test on autologous sections of gastric mucosa. No antigen retrieval was performed, to avoid any background staining due to detection of endogenous immunoglobulins.

The 4-μm-thick sections were dewaxed in xylene, re-hydrated through graded ethanol to distilled water. Endogenous peroxidase in tissues was blocked with pre-incubation for 30 min in 0.3% H_2_O_2_ (v/v) in methanol followed by washing with distilled water. All subsequent incubations were performed at room temperature. Non-specific binding of human serum immunoglobulins was blocked with pre-incubation for 30 min in PBS-30% normal rabbit serum (NRS). The sections were then incubated for 2 h with patient's serum diluted 1:20 in PBS-30% NRS. After three washes with PBS for 3 min each, peroxidase-coupled rabbit anti-human IgG antibody (Dako) diluted 1:200 was applied for 45 min. After washing, the antigen-antibody binding was visualized with 3.3′-diaminobenzidine application (DAB; Dako). The sections were then counterstained with hematoxylin, dehydrated, and coverslipped.

The immunohistochemical staining for monoclonal antibodies was performed with the antibodies at a concentration of 5 μg/ml, using a streptavidin-biotin-peroxidase detection system (Biogenex).

### Preabsorption of Sera

An aliquot of bacterial suspension was centrifuged on a microfuge at 11,000 rpm for 2 min. The supernatant was discarded and the bacterial pellet obtained was re-suspended in 10 μl of patient's serum sample. The suspension was vortexed 5 times for 10 s each, with a 5-min interval between each vortexing, to favor the immunoadsorption of antibodies to bacterial cells. The suspension was further added with 190 μl of serum dilution buffer, vortexed and centrifuged at 11,000 rpm for 2 min. The supernatant preabsorbed patient's serum was then used as primary antibody in coupled repeated immunohistochemistry as described above to assess if any residual reactivity toward gastric autoantigens was present.

### *H. pylori* Typing With Monoclonal Antibodies

An aliquot of bacterial suspension was diluted in distilled water, distributed in microwells (100 μl/well) and allowed to dry overnight at 37°C. The wells were washed, overcoated with 5% normal rabbit serum for 60 min and then incubated 60 min. with each monoclonal antibody (5 μg/ml in PBS-1% BSA). Peroxidase-labeled rabbit anti-mouse immunoglobulin was used as the second antibody. After washing, the reactions were developed with TMB substrate and read at 450 nm.

### Western Blotting

A mixture of *H. pylori* cells derived from two patients with a previous history of myocardial infarction was diluted in SDS buffer (10^9^ cells/ml), heated for 5 min at 100°C, run on a on a single-well 10% polyacrilamide gel and transferred to nitrocellulose.

After blocking with 3% BSA, the sheets were incubated with human test sera (1:30) or monoclonal antibodies (5 μg/ml) diluted in PBS with 5% normal rabbit serum for 60 min at 37°C. After being washed with PBS-0.05 Tween 20, the strips were incubated for 1 h at 37°C with peroxidase conjugated rabbit anti human or anti mouse total Ig. After three washing steps, the antigen-antibody binding was highlighted with 3.3′-diaminobenzidine chromogen application.

### Agglutination Test

Blood samples from healthy donors were collected into tubes containing EDTA. A drop of 3% suspension of red blood cells in PBS and a drop of anti LeY IgM monoclonal antibody 1E52 (100 μg/ml in PBS) were added to a test tube, mixed and incubated at room temperature for 15 min. After centrifugation for 10 s at 800 rpm, the red blood cell button was dislodged and observed for agglutination.

## Results

### Histological Findings in Mice and Rabbits

Five mice of the *H. pylori* immunized group were found dead, without any prior clinical sign, during the immunization period, between 30 and 60 days after the first burst. Only one mouse of the control group died (Table 1). We considered the initial few deaths as non-significant events. Therefore, none of these dead mice was subjected to necropsy.

**Table 1:** Histological analysis of hearts of mice and rabbits after immunization with *H. pylori*.

	**Immunization**	**Total**	**Found dead**	**Histologically examined**	**Myocardial necrosis**
**MICE**					
Tests	*H. pylori*	38	5	20	5
Controls	*C. jejuni*	24	0	20	0
	Pool of gram neg bact	2	0	2	0
	not immunized	8	1	8	0
	TOTAL CONTROLS	34	1	30	0
**RABBITS**					
Tests	*H. pylori*	5	0	5	2
Controls	*C. jejuni*	3	0	3	0
	Not immunized	3	0	3	0
	Total Controls	6	0	6	0

*The controls were from animals belonging to the same lot, immunized with C. jejuni, a pool of gram negative bacteria or not immunized. The six mice found dead in cages were not examined histologically, because the decision to keep the organs was taken later, after the observation that many of the anti H. pylori monoclonal antibodies obtained cross-reacted with gastric and extra-gastric tissues*.

After we found that more than 30% of the anti *H. pylori* monoclonal antibodies obtained from these mice were also autoantibodies reacting with gastric and extra-gastric epithelia, we decided to keep the organs in order to study, at a later time, other possible extra-gastric effects of the cross-reactive autoimmune response.

**Figure 2:**
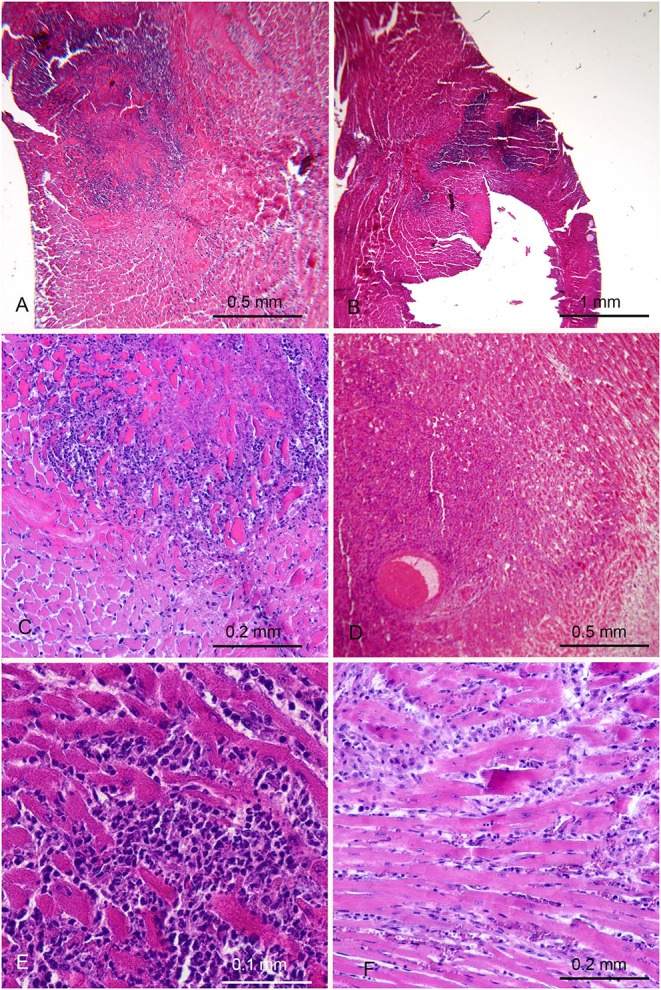
Myocardial lesions in mice after parenteral immunization with *H. pylori* cells occurred months earlier. **(A,B)** Low magnification images in two mice showing extensive areas suggestive of recent myocardial infarction. **(C)** Higher magnification showing a strong granulocytic infiltration among the myocytes involved in coagulative necrosis. **(D)** Thrombosis of an intramural arterial vessel, embedded in a necrotic area with heavy polymorphonuclear and mononuclear infiltration. **(E,F)** In other 2 mice small areas of recent myocyte necrosis were only found, with variable degree of interstitial oedema and infiltration by polymorphonuclear and mononuclear leukocytes.

The thorough histological analysis of the 20 mice immunized with *H. pylori* examined (Table 1) showed well-circumscribed areas of myocardial necrosis in 5 of them (Figure 2). In two of these 5 mice the necrotic area was large, with a maximum dimension of ~1 mm. The changes observed appeared very similar to those described in the different phases of myocardial infarction in humans. The necrotic areas showed karyorrhexis and disintegration of myofibrils with associated dense neutrophilic infiltration and macrophage phagocytosis (Figures 2A–C), suggesting a recent infarction (<15 days). In one mouse the myocardial lesions were associated with thrombosis in intramural arterial segments (Figure 2D). These 2 mice were killed, respectively, 3 and 5 months after the first burst because of their emaciated appearance. The three remaining mice showed smaller foci of necrosis associated with interstitial inflammation (Figures 2E,F). They appeared asymptomatic and were killed at the end of the study, ~12 months after the first burst.

The hearts of control mice, including the one found dead (Table 1), did not show any significant histological change.

The association between presence of myocardial necrosis and immunization with *H. pylori* was statistically significant according to the chi-square test (*p* < 0.001)

Two of the five rabbits immunized with *H. pylori* (Table 1) showed extensive myocardial infarct with different phase of evolution. The maximum dimension of the infarctions measured histologically were ~5 mm.

**Figure 3:**
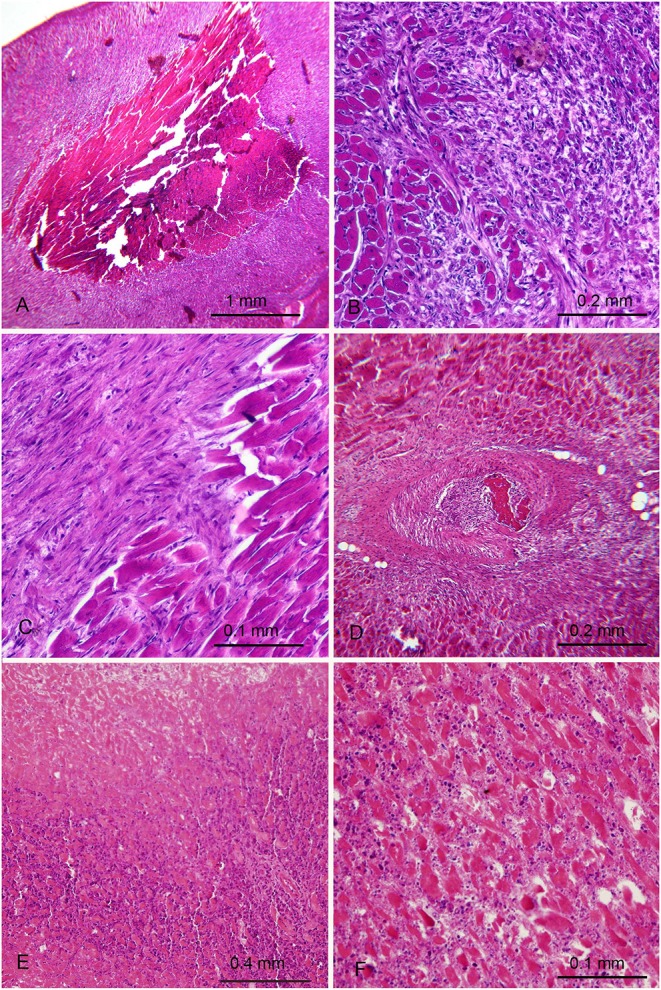
Myocardial lesions in rabbits after parenteral immunization with *H. pylori* cells occurred months earlier. **(A–D)** First rabbit with old myocardial infarction with a necrotic mummified core **(A)** demarcated by granulation tissue **(B)** with partial transformation in fibrosis **(C)** and presence of a thrombotic arterial occlusion undergoing organization **(D)**. **(E,F)** Second rabbit with more recent infarction. **(E)** necrotic area (top) surrounded by a dense neutrophilic infiltrate **(F)** A different zone at higher magnification showing eosinophilic necrotic myocytes, edema and significant polymorphonuclear infiltrate.

In the first animal (Figures 3A–D) an injury probably older than 1 month was seen. It was characterized by a large core of mummified myocytes with a wide circumferential zone of florid granulation tissue turning into fibrosis. A coronary occluding thrombus undergoing organization was also found Figure 3D. This rabbit appeared asymptomatic and was killed at the end of the study, at the age of 16 months, 4 months after the last burst was delivered. The myocardial infarction of the second rabbit was more recent and showed coagulative necrosis, interstitial oedema and dense neutrophilic infiltrate (Figures 3E,F). This second rabbit was killed 48 days after the last burst, at the age of 7 months, because of its emaciated appearance. The hearts of the 6 control rabbits did not show any significant histological change. The association between infarction and *H. pylori* immunization was noteworthy though not statistically significant, most likely because of the low number of rabbits involved.

### Immunohistochemical Comparison of Human and Mouse Autoantibodies Induced by *H. pylori*

**Figure 4:**
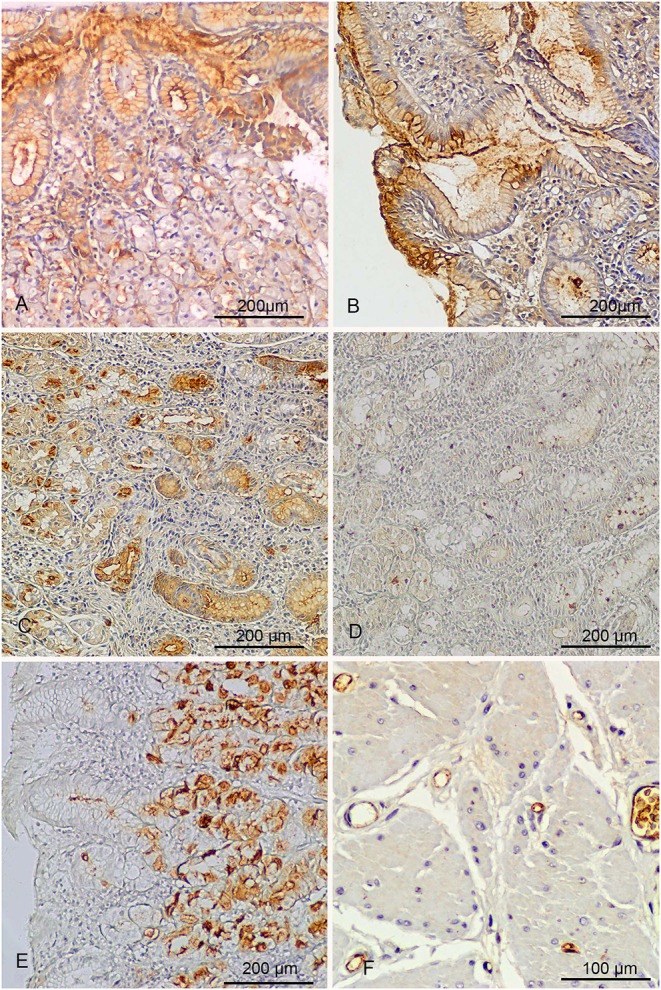
Immunohistochemical analysis. **(A)** The serum of a patient with *H. pylori* gastritis and previous history of myocardial infarction tested against autologous gastric biopsy for the presence of autoantibodies. Positive reaction with gastric mucins at the surface and within the cytoplasm of foveolar epithelium (brown staining). **(B)** Reactivity similar to that observed in patient of A, obtained with a monoclonal antibody cross-reacting with H type 1 (4D2). **(C)** The strong autoantibody activity against cytoplasmic mucin of both superficial and glandular gastric epithelium in second patient with *H. pylori* and previous history of myocardial infarction. Note also the reaction against the canalicular structures of parietal cells (left). **(D)** Complete neutralization of autoantibodies obtained with pre-absorption of serum with *H. Pylori*. **(E)** The reaction of an anti *H. pylori* monoclonal antibody cross-reacting with LeY (1e52), concentrated in the glandular portion of gastric epithelium. **(F)** Strong expression of LeY at the surface of red blood cells and endothelium, detected with antibody 1E52.

When tested on sections of autologous gastric biopsies, the sera from the two patients with previous history of myocardial infarction showed a strong autoantibody reactivity against mucins at the surface of and within gastric epithelial cells (Figures 4A,C).

The specificity of this reaction was proven by the absence of reactivity when sections of the same gastric biopsies were tested with sera from *H. pylori* negative patients (negative control).

In the first patient the autoantibodies reacted mainly with the surface epithelium (Figure 4A). The reaction beared similarities with that of monoclonal antibodies cross-reacting with H type 1 and LeB (Figure 4B), consistent with predominant expression of type 1 chains by the superficial epithelium. The preincubation of gastric mucosal sections with a high concentration of antibody 4D2, specific for H-type1, produced a partial inhibition of this autoantibody reaction.

The second patient showed a more severe chronic gastritis and a stronger autoantibody reaction involving both the surface and glandular epithelium (Figure 4C). The autoantibodies also reacted with the canalicular membrane of parietal cells, the site of H + /K + -ATPase, the gastric proton pump.

The cross-reacting nature of the autoantibodies present in these two patients was demonstrated by their neutralization after pre-absorption of sera with a pool of *H. pylori* strains (Figure 4D).

In both patients the monoclonal anti-*H. pylori* antibodies cross-reacting with LeY and LeX reacted mainly with the glandular epithelium, known to display mainly H type 2 chains (Figure 4E).

The observation that two of the three anti LeY antibodies (CB-4 and 1E52) weakly stained some of the few red cells present in the biopsy samples prompted us to test all three antibodies against some archival normal tissue samples.

CB-4 and 1E52 strongly immunostained the luminal endothelial membrane and the surface of red cells in 5 out of the 6 samples tested (Figure 4F). No staining was observed with antibody (HpN35).

### Agglutination Test

To further evaluate the frequency of the expression of LeY in endothelium and red cells in normal individuals we tested antibody 1E52 (IgM) with a small panel of blood samples from blood donors by a direct hemagglutination test. As shown in Table 2, all group O samples were strongly positive, in contrast with group A samples, 3 of which only were weakly positive.

**Table 2:** Frequency of LeY expression in blood donors detected with monoclonal anti *H. pylori* antibody 1E52 through a direct hemagglutination test.

	**N**°****	**Le Y positive**	**%**
Group O	15	15	100
Group A	15	3	20
Group B	4	0	0

### Reactivity of Monoclonal Antibodies With Synthetic Antigens

Reactivity is shown in Table 3. The antibodies appear to be highly specific for each glycoconjugate antigens, as reported in part in previous studies (12). In particular, none of the anti LeY antibodies cross-reacted with blood group H (especially H type 2), typically present on red blood cells.

**Table 3:** Summary of binding specificities of *H. pylori*-induced autoreactive Mabs with synthetic Lewis antigens.

	**Le X**	**Le Y**	**H type 1**	**H type 2**	**Le A**	**Le B**	**HSA*^b^***	**BSA*^b^***
CB-10	+++*^a^*	–	–	–	–	–	–	–
1E52, CB-4, HpN35	-	+++	-	-	-	–	–	–
4D2	–	–	+++	–	–	–	–	–
19-OLE*^c^*	–	++	–	+++	–	–	–	–

a *ELISA results were scored as: –, < 0.1; +,0.1– < 0.5; ++, 0.5–1.0; +++, >1.0*.

b *Bovine (BSA) and human (HSA) serum albumins*.

c *19-OLE, a control antibody anti-H type 2. This antibody appears to exhibit some cross reactivity with Lewis Y*.

### Antigenic Profile of *H. pylori* Isolates

**Figure 5:**
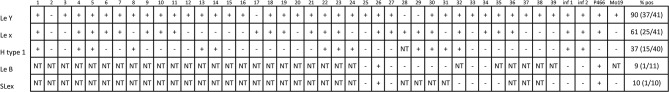
Expression of blood group related antigens by 41 *H. pylori* clinical isolates as detected by a panel of monoclonal antibodies. A and B = *H. pylori* strains from the two patients with previous myocardial infarction. NT, not tested. P466 and Mo19 = two reference *H. pylori* strains.

The expression of histo-blood group related antigens by a panel of 41 clinically isolated *H. pylori* strains, including those from the two patients with previous history of myocardial infarction, has been tested by monoclonal antibodies (Figure 5). The frequency of expression of LeY, LeX, H type 1, LeB and Sialyl LeX was, respectively, of 90, 61, 37, 9, and 10%. The reactivity of the two control strains P466 and Mo19 is also shown in Figure 5. The infecting *H. pylori* isolates from the 2 patients with previous history of myocardial infarction expressed LeX, LeY, H type 1, but not LeB nor Sialyl-LeX (Figure 5).

### Human and Mouse Autoantibodies Induced by *H. pylori* Analyzed by Western Blotting

The sera of the two patients with previous myocardial infarction, and, for comparative purpose, 30 randomly selected sera from patients previously undergoing gastroscopy (29 found *H. pylori* positive and one negative as a negative control) were studied by immunoblot analysis. A whole cell bacterial mixture of the H. pylori clinical isolates from the two patients with myocardial infarction was used as the antigen.

The sera exhibited a heterogeneous IgG reaction to numerous bands with variable intensity. Anti *H. pylori* monoclonal antibodies cross-reacting with histo-blood group antigens reacted with a few bands between 30 and 45 kDa. These bands were also recognized by the sera of both patients with previous myocardial infarction as well as by most sera of the other patients. This is consistent with the previous finding that most patients with the infection have autoantibodies induced by antigenic mimicry with host structures by LPS.

## Discussion

Several mechanisms have been proposed to explain the link between *H. pylori* and cardiovascular diseases, including platelet aggregation, increased homocysteine blood levels, production of proinflammatory cytokines and an abnormal immune response (21).

In this retrospective study we provide the first experimental evidence suggesting that the immune response against *H. pylori* may induce myocardial infarction in animals.

The careful histological examination of hearts from mice and rabbits immunized with *H. pylori* in our previous studies shows the presence of extensive necrotic lesions in some of them. No similar lesions were found in control animals, suggesting a causal link with *H. pylori* immunization.

The hearts of the animals used in our experimental studies had not been extensively examined previously, because the role of *H. pylori* in cardiovascular disease had not yet emerged at the time of our studies. At the beginning, we considered the observed deaths in cages as non-significant events. After we found that more than 30% of the anti *H. pylori* monoclonal antibodies obtained from mice immunized with *H. pylori* were also autoantibodies reacting with extra-gastric epithelia, we began archiving the organs of the animals involved in our experiments, without analyzing them, with a view to future studies.

We did not perform any postmortem examination of the first five *H. pylori* immunized mice found dead. In hindsight, the finding that five other immunized mice developed myocardial infarction leads us to consider this disease as the most probable cause for these sudden deaths. The fact that the animals were not infected but only previously immunized with parenteral injections of dead *H. pylori* cells, strongly suggests that the myocardial lesions were a consequence of the immunization.

In some of the animals, myocardial infarction was associated with coronary thrombotic occlusion in the absence of signs of background atherosclerosis. We propose that the immune response against *H. pylori* may have resulted in a thrombotic occlusion either via direct endothelial damage or by perturbation of the coagulation process or through both these mechanisms.

The high tendency to develop myocardial lesions observed in our animals may be due to the high degree of immunization induced by repeated subcutaneous injection of bacterial suspensions or to the presence, within the pool of *H. pylori* isolates used for immunization, of a highly pathogenic strain.

Both patients with previous history of myocardial infarction exhibited autoantibodies directed against mucins of autologous gastric epithelium, demonstrated by a strong immunohistochemical reaction, similar to what we have previously described in patients with *H. pylori* infection. These autoantibodies were also anti *H. pylori* antibodies recognizing epitopes shared with host antigenic components (antigenic mimicry). This was clearly demonstrated by neutralization of their binding with gastric epithelium obtained with pre-absorption of sera with *H. pylori* cells.

Mucins are particularly rich in glycan (carbohydrate) structures and express blood group ABO and Lewis-type antigens (25). Glycans decorate the surfaces of all prokaryotic and eukaryotic cells and viruses. Anti-glycan antibodies constitute a large fraction of whole human serum immunoglobulins (26). The best known anti-glycan antibodies are those directed against blood group A and B alloantigens in human plasma. It is commonly believed that these antibodies may be, at least in part, produced in response to bacterial antigens expressed by gut microbiota (27). When the immune cross-reaction involves self-antigens the antibodies become true, potentially pathogenic, autoantibodies. The issue is to establish how anti glycan autoantibodies induced by *H. pylori* may be involved in providing the link between *H. pylori* and cardiovascular disease.

Most of our cross-reacting monoclonal antibodies recognized LeX, LeY, and H type 1 (10, 12), but the specificity of other cross-reacting antibodies produced by us has not been identified.

*H. pylori* may also produce other blood group related autoantigenic targets, like LeA, LeB, non fucosylated type 2 precursor (i-antigen), non-fucosylated type 1 precursor (LeC) (Figure 1), Thomsen-Friedenreich antigen, blood group A (28–31). There are probably further not yet identified antigens.

The *H. pylori* strains isolated by the two patients with previous myocardial infarction were positive for LeX, LeY, and H type 1.

LeX, also called CD15 or SSEA-1, is expressed on mature myeloid cells and is associated with chemotaxis, phagocytosis and cell adhesion. Our *H. pylori*-induced anti-LeX monoclonal antibodies revealed that LeX is also expressed by most strains of *H. pylori* as well as by mucins of the glandular epithelium of the stomach. LeX is not expressed by endothelial cells, but interestingly, using immunoaffinity chromatography, it has been shown that it, as well as sialyl-LeX, are present in many plasma glycoproteins (32, 33).

Sialyl-LeX (SLeX or CD15s), a well-known glycan molecule involved in endothelium-leukocyte adhesion, is another epitope that may be expressed by some *H. pylori* strains (34). *H. pylori* strains isolated from both patients with previous myocardial infarction did not express Sialyl-LeX, suggesting that it is not essential in the pathogenesis of myocardial injury.

LeY (CD174) is restrictedly expressed at the apical surface of some epithelial cells (35, 36). Our monoclonal antibodies revealed that, like LeX, LeY too is strongly expressed in gastric epithelial mucins (8–10, 12). We previously reported that mice bearing hybridomas secreting an anti LeY cross-reacting antibody (CB-4) developed gastric cytotoxic effects *in vivo*, indicating a direct pathogenic role for anti- Lewis antibodies (9). LeY is not expressed in endothelial cells of mice and rabbits. Should the strong anti LeY autoantibody reaction induced by immunization with *H. pylori* seen in animals be involved in determining their myocardial lesions, these antibodies would therefore exert their pathogenic effect by reacting with LeY expressed in circulating glycoproteins or glycolipids, rather than in endothelium. In humans, instead, Lin et al. recently reported that LeY is barely detectable in carotid arteries while its expression increases after carotid ligation (37). The authors also provide evidences that LeY is a carbohydrate induced by inflammation on endothelial cells and it mediates leukocyte adhesion. Interestingly, one of our anti LeY antibodies (1E52) strongly reacted not only with endothelium, but also with erythrocytes of all blood group O individuals. Subjects with blood groups A or B were mostly negative, most probably because of competition between A, B and Lewis enzymes for their common precursor, the H antigen (type 2). In this regard, antibody 1E52 doesn't cross-react with blood group H (especially H type 2), which is a substructure of LeY and is typically present on red blood cells. Therefore, it can be excluded that the reactivity of 1E52 could be the result of binding to both Lewis Y and H antigen.

Circulating autoantibodies anti-LeY were barely or inconstantly detected in patients with *H. pylori* infection (38). This could be due to the technological problem of using synthetic antigens to detect anti-glycan antibodies (12, 39). In addition, a portion of a pathogenic autoantibody might be undetectable because already immunocomplexed *in vivo* with the corresponding cell surface or soluble host autoantigens. If we assume that in humans *H. pylori* induces anti LeY antibodies of pathogenic potential for endothelium, in group O individuals the overwhelming majority of these antibodies would be immediately neutralized because adsorbed onto LeY expressed in large amount in erythrocytes. Conversely, in group A and B individuals, these autoantibodies would be able to react with endothelial LeY epitopes unmasked by a local endothelial-cell injury. This hypothesis is consistent with the recent finding by Groot et al. (40) that, blood groups A and B are associated with increased risk for thromboembolic events, including myocardial infarction, if compared with blood group O.

The *H. pylori* strains isolated from both patients with myocardial infarct expressed H type 1 antigen. The autoantibody reaction in both patients resembled that obtained with anti *H. pylori* monoclonal antibody recognizing H type 1(4D2), suggesting that, at least in part, the autoimmune reaction involves type 1 chain derived antigens. These antigens have been described to be absent or hardly detectable in mouse and rabbit species (41, 42). However, we found that, as for LeX and LeY, H type 1 appear strongly expressed in mouse gastric mucins when detected by anti- *H. pylori* monoclonal antibodies cross-reacting with histo-blood group antigens (9, 10, 43). H type 2 is not expressed by *H. pylori* (44).

Type 1 chain derived histo-blood group like antigens are synthesized by endodermal epithelia, such as gut epithelial cells, but not by endothelial and erythroid cells. They are variable expressed on plasma glycoproteins and glycolipids and are also absorbed onto the erythrocyte membrane (45, 46).

It cannot be excluded that certain anti *H. pylori* antibodies could cross react with glycans attached to lipids (glycolipids) and proteins (glycoproteins), so favoring an acquired hypercoagulable state. In relation to this, one of the most important markers of acquired thrombophilia is the presence of antibodies against beta-2 glycoprotein (B2GP). This protein is highly glycosylated. Antibodies against B2GPI are present in 3–5% of the general population (47). Interestingly, a study on a child population has shown that *H. pylori* is associated to the presence of circulating anti-cardiolipin/B2GPI and that *H. pylori* eradication leads to their disappearance (48). Other circulating glycoproteins involved in the coagulation process and expressing blood group-related molecules and their precursor antigens are von Willebrand factor (49, 50), coagulation factor VIII (51, 52). and a portion of α(2)-macroglobulin (α(2)M) (53).

The existence of antibodies against gastric mucins is a clear demonstration that *H. pylori* can induce a strong autoimmune response. However, real antigenic specificity of cross-reacting antibodies possibly promoting the development of the myocardial lesions observed in our animals remains to be determined.

The major limitation of this study is that the data have been obtained in experimental animals only and cannot be directly extrapolated to humans. To assess the possible role of *H. pylori* induced autoimmunity as one of the risk factors for myocardial infarction in humans through our suggested mechanism, further studies are needed involving a large number of *H. pylori* strains and sera from myocardial infarction patients.

A further limitation of this study is represented by the small number of control mice immunized with a pool of gram-negative bacteria. It would be interesting to assess the specificity of *H. pylori* induced autoantibody production against a larger number of mice immunized with a pool of gram-negative bacteria. In conclusion, this retrospective study suggests that *H. pylori* induces autoantibodies possibly involved in causing myocardial infarction in mice and rabbits and provides a useful experimental model for the understanding the real importance of *H. pylori* antigenic mimicry in the pathogenesis of cardiovascular diseases.

## Data Availability Statement

All datasets generated for this study are included in the article/supplementary material.

## Ethics Statement

The Ethic Committee of Spedali Civili—Brescia (Italy) certifies that this study does not require any ethical approval under Italian Law, since it is a retrospective evaluation using human and animal materials archived during previous published experimental studies (8–10, 24). All experiments were performed in accordance with relevant guidelines and regulations. All the human patient data/samples were fully anonymized before we accessed them and the requirement for informed consent from these patients was therefore considered unnecessary.

## Author Contributions

RN conducted experiments, performed histological analyses, and wrote manuscript. VV performed histological analyses. CP conducted experiments and analyzed data. AS performed histological analyses and revised the manuscript. All authors have read and approved the manuscript as submitted.

### Conflict of Interest

The authors declare that the research was conducted in the absence of any commercial or financial relationships that could be construed as a potential conflict of interest.

## References

[1] Ross R. Atherosclerosis–an inflammatory disease. N Engl J Med. (1999) 340:115–26. 10.1056/nejm199901143400207 9887164

[2] Wolf D Ley K. Immunity and inflammation in atherosclerosis. Circ Res. (2019) 124:315–27. 10.1161/circresaha.118.313591 30653442 PMC6342482

[3] Campbell LA Kuo CC *Chlamydia pneumoniae*–an infectious risk factor for atherosclerosis? Nat Rev Microbiol. (2004) 2:23–32. 10.1038/nrmicro796 15035006

[4] Liu J Wang F Shi S. *Helicobacter pylori* infection increase the risk of myocardial infarction: a meta-analysis of 26 studies involving more than 20,000 participants. Helicobacter. (2015) 20:176–83. 10.1111/hel.12188 25382293

[5] Lee M Baek H Park JS Kim S Kyung C Baik SJ. Current *Helicobacter pylori* infection is significantly associated with subclinical coronary atherosclerosis in healthy subjects: a cross-sectional study. PLoS ONE. (2018) 13:e0193646. 10.1371/journal.pone.0193646 29499055 PMC5834174

[6] Jia YJ Liu J Han FF Wan ZR Gong LL Liu H. Cytomegalovirus infection and atherosclerosis risk: a meta-analysis. J Med Virol. (2017) 89:2196–206. 10.1002/jmv.24858 28513970

[7] Matsuura E Atzeni F Sarzi-Puttini P Turiel M Lopez LR Nurmohamed MT. Is atherosclerosis an autoimmune disease? BMC Med. (2014) 12:47. 10.1186/1741-7015-12-47 24642015 PMC3984678

[8] Negrini R Lisato L Cavazzini L Maini P Gullini S Basso O. Monoclonal antibodies for specific immunoperoxidase detection of *Campylobacter pylori*. Gastroenterology. (1989) 96:414–20. 10.1016/0016-5085(89)91565-5 2642876

[9] Negrini R Lisato L Zanella I Cavazzini L Gullini S Villanacci V. *Helicobacter pylori* infection induces antibodies cross-reacting with human gastric mucosa. Gastroenterology. (1991) 101:437–45. 10.1016/0016-5085(91)90023-E 2065920

[10] Negrini R Savio A Poiesi C Appelmelk BJ Buffoli F Paterlini A. Antigenic mimicry between Helicobacter pylori and gastric mucosa in the pathogenesis of body atrophic gastritis. Gastroenterology. (1996) 111:655–65. 10.1053/gast.1996.v111.pm8780570 8780570

[11] Rojas M Restrepo-Jiménez P Monsalve DM Pacheco Y Acosta-Ampudia Y Ramírez-Santana C. Molecular mimicry and autoimmunity. J Autoimmun. (2018) 95:100–23. 10.1016/j.jaut.2018.10.012 30509385

[12] Appelmelk BJ Simoons-Smit I Negrini R Moran AP Aspinall GO Forte JG. Potential role of molecular mimicry between *Helicobacter pylori* lipopolysaccharide and host Lewis blood group antigens in autoimmunity. Infect Immun. (1996) 64:2031–40. 8675304 10.1128/iai.64.6.2031-2040.1996 PMC174033

[13] Li H Tang H Debowski AW Stubbs KA Marshall BJ Benghezal M. Lipopolysaccharide structural differences between western and asian helicobacter pylori strains. Toxins. (2018) 10:364. 10.3390/toxins10090364 30205541 PMC6162551

[14] Stanley P Cummings RD Structures common to different glycans. In: Varki A Cummings RD Esko JD Stanley P Hart GW Aebi M Darvill AG Kinoshita T Packer NH Prestegard JH Schnaar RL Seeberger PH editors Essentials of Glycobiology. 3rd Edn, New York, NY: Cold Spring Harbor Laboratory Press (2017), p. 161–78.

[15] Daniels G Abo H Lewis systems In: Daniels G editor. Human Blood Groups, 3rd Edn. (Chicester: Wiley-Blackwell) (2013), p. 11–95.

[16] Aspinall GO Monteiro MA Pang H Walsh EJ Moran AP. Lipopolysaccharide of the Helicobacter pylori type strain NCTC 11637 (ATCC 43504): structure of the O antigen chain and core oligosaccharide regions. Biochemistry. (1996) 35:2489–97. 10.1021/bi951852s 8652593

[17] Aspinall GO Monteiro MA. Lipopolysaccharides of Helicobacter pylori strains P466 and MO19: structures of the O antigen and core oligosaccharide regions. Biochemistry. (1996) 35:2498–504. 10.1021/bi951853k 8652594

[18] RaŽuka-Ebela D Giupponi B Franceschi F. Helicobacter pylori and extragastric diseases. Helicobacter. (2018) 23 (Suppl. 1):e12520. 10.1111/hel.12520 30203590

[19] Chey WD Leontiadis GI Howden CW Moss SF. ACG clinical guideline: treatment of *Helicobacter pylori* infection. Am J Gastroenterol. (2017) 112:212–39. 10.1038/ajg.2016.563 28071659

[20] Neunert C Lim W Crowther M Cohen A Solberg L Crowther MA. The American Society of Hematology 2011 evidence-based practice guideline for immune thrombocytopenia. Blood. (2011) 117:4190–207. 10.1182/blood-2010-08-302984 21325604

[21] Chmiela M Gajewski A Rudnicka K. *Helicobacter pylori* vs coronary heart disease - searching for connections. World J Cardiol. (2015) 7:187–203. 10.4330/wjc.v7.i4.187 25914788 PMC4404374

[22] Yu XJ Yang X Feng L Wang LL Dong QJ. Association between *Helicobacter pylori* infection and angiographically demonstrated coronary artery disease: a meta-analysis. Exp Ther Med. (2017) 13:787–93. 10.3892/etm.2017.4028 28352367 PMC5348668

[23] Wang JW Tseng KL Hsu CN Liang CM Tai WC Ku MK. Association between *Helicobacter pylori* eradication and the risk of coronary heart diseases. PLoS ONE. (2018) 13:e0190219. 10.1371/journal.pone.0190219 29293574 PMC5749777

[24] Negrini R Zanella I Poiesi AS Verardi R Ghielmi S Albertini A. Serodiagnosis of helicobacter pylori-associated gastritis with a monoclonal antibody competitive enzyme-linked immunosorbent assay. Scand J Gastroenterol. (1992) 27:599–605. 10.3109/00365529209000125 1641587

[25] Schenkel-Brunner H ABO(H) system. In: Schenkel-Brunner H editor. Human Blood Groups: Chemical and Biochemical Basis of Antigen Specificity. Berlin: Springer Science & Business Media (2013). p. 47–145.

[26] Durbin SV Wright WS Gildersleeve JC. Development of a multiplex glycan microarray assay and comparative analysis of human serum anti-glycan IgA, IgG, and IgM repertoires. ACS Omega. (2018) 3:16882–91. 10.1021/acsomega.8b02238 30613809 PMC6312630

[27] Donald B Anti-A and anti-B: what are they and where do they come from? Transfusion. (2015) 55 (Suppl 2):S74–9. 10.1111/trf.13087 26174901

[28] Monteiro MA Zheng P Ho B Yokota S Amano K Pan Z. Expression of histo-blood group antigens by lipopolysaccharides of *Helicobacter pylori* strains from Asian hosts: the propensity to express type 1 blood-group antigens. Glycobiology. (2000) 10:701–13. 10.1093/glycob/10.7.701 10910974

[29] Monteiro MA Chan KH Rasko DA Taylor DE Zheng PY Appelmelk BJ. Simultaneous expression of type 1 and type 2 lewis blood group antigens by *Helicobacter pylori* lipopolysaccharides. J Biol Chem. (1998) 273:11533–43. 10.1074/jbc.273.19.11533 9565568

[30] Klaamas K Kurtenkov O Rittenhouse-Olson K Brjalin V Miljukhina L Shljapnikova L. Expression of tumor-associated Thomsen-Friedenreich antigen (T Ag) in Helicobacter pylori and modulation of T Ag specific immune response in infected individuals. Immunol Invest. (2002) 31:191–204. 10.1081/imm-120016240 12472179

[31] Heneghan MA McCarthy CF Moran AP. Relationship of blood group determinants on *Helicobacter pylori* lipopolysaccharide with host lewis phenotype and inflammatory response. Infect Immun. (2000) 68:937–41. 10.1128/iai.68.2.937-941.2000 10639467 PMC97226

[32] Cho W Jung K Regnier FE. Use of glycan targeting antibodies to identify cancer-associated glycoproteins in plasma of breast cancer patients. Anal Chem. (2008) 80:5286–92. 10.1021/ac8008675 18558770

[33] Cho W Regnier FE Jung K. Sialylated Lewis x antigen bearing glycoproteins in human plasma. J Proteome Res. (2010) 9: 5960–8. 10.1021/pr100747p 20858014 PMC2976037

[34] Monteiro MA Appelmelk BJ Rasko DA Moran AP Hynes SO MacLean LL. Lipopolysaccharide structures of *Helicobacter pylori* genomic strains 26695 and J99, mouse model *H. pylori* Sydney strain, *H. pylori* P466 carrying sialyl Lewis X, and H. pylori UA915 expressing Lewis B. Eur J Biochem. (2000) 267:305–20. 10.1046/j.1432-1327.2000.01007.x 10632700

[35] Mollicone R Bara J Le Pendu J Oriol R. Immunohistologic pattern of type 1 (Lea, Leb) and type 2 (X, Y, H) blood group-related antigens in the human pyloric and duodenal mucosae. Lab Invest. (1985) 53:219–27. 2410664

[36] Croce MV Isla-Larrain M Rabassa ME Demichelis S Colussi AG Crespo M. Lewis x is highly expressed in normal tissues: a comparative immunohistochemical study and literature revision. Pathol Oncol Res. (2007) 13:130–8. 10.1007/bf02893488 17607374

[37] Lin W Chang C Shi C Shi G Wu H. Recombinant lectin-like domain of thrombomodulin suppresses vascular inflammation by reducing leukocyte recruitment via interacting with lewis Y on endothelial cells. Arterioscler Thromb Vasc Biol. (2013) 33:2366–73. 10.1161/atvbaha.113.301221 23950139

[38] Chmiela M Gonciarz W. Molecular mimicry in *Helicobacter pylori* infections. World J Gastroenterol. (2017) 23:3964–77. 10.3748/wjg.v23.i22.3964 28652651 PMC5473117

[39] Heneghan MA McCarthy CF Janulaityte D Moran AP. Relationship of anti-Lewis x and anti-Lewis y antibodies in serum samples from gastric cancer and chronic gastritis patients to Helicobacter pylori-mediated autoimmunity. Infect Immun. (2001) 69:4774–81. 10.1128/iai.69.8.4774-4781.2001 11447150 PMC98564

[40] Groot HE Villegas Sierra LE Said MA Lipsic E Karper JC van der Harst P. Genetically determined ABO blood group and its associations with health and disease. Arterioscler Thromb Vasc Biol. (2020) 40:830–8. 10.1161/ATVBAHA.119.313658 31969017

[41] Magalhães A Gomes J Ismail MN Haslam SM Mendes N Osório H. Fut2-null mice display an altered glycosylation profile and impaired BabA-mediated Helicobacter pylori adhesion to gastric mucosa. Glycobiology. (2009) 19:1525–36. 10.1093/glycob/cwp131 19706747 PMC2782244

[42] Nyström K Le Gall-Reculé G Grassi P Abrantes J Ruvoën-Clouet N Le Moullac-Vaidye B. Histo-blood group antigens act as attachment factors of rabbit hemorrhagic disease virus infection in a virus strain-dependent manner. PLoS Pathog. (2011) 7:e1002188. 10.1371/journal.ppat.1002188 21901093 PMC3161982

[43] Simoons-Smit IM Appelmelk BJ Verboom T Negrini R Penner JL Aspinall GO. Typing of Helicobacter pylori with monoclonal antibodies against Lewis antigens in lipopolysaccharide. J Clin Microbiol. (1996) 34:2196–200. 8862584 10.1128/jcm.34.9.2196-2200.1996 PMC229216

[44] Wang G Cha NW Boulton PG Taylor DE Palcic MM. Novel *Helicobacter pylori* α1,2-fucosyltransferase, a key enzyme in the synthesis of Lewis antigens. Microbiology. (1999) 145:3245–53. 10.1099/00221287-145-11-3245 10589734

[45] Oriol R Le Pendu L Mollicone R. Genetics of ABO, H, Lewis, X and related antigens. Vox Sang. (1986) 51:161–71. 10.1111/j.1423-0410.1986.tb01946.x 2433836

[46] Henry SM. Review: phenotyping for Lewis and secretor histo-blood group antigens. Immunohematology. (1999) 12:51–61. 15387741

[47] De Groot PG Urbanu RT The significance of autoantibodies against 2-glycoprotein I. Blood. (2012) 120:266–74. 10.1182/blood-2012-03-378646 22553312

[48] Sarici SU Gursel O Kurekci E Kesik V Atay A Okutan V. Anticardiolipin antibodies in children with *Helicobacter pylori* infection. Helicobacter. (2015) 20:418–21. 10.1111/hel.12226 25856798

[49] Canis K Mckinnon TA Nowak A Panico M Morris HR Laffan M. The plasma von Willebrand factor O-glycome comprises a surprising variety of structures including ABH antigens and disialosyl motifs. J Thromb Haemost. (2010) 8:137–45. 10.1111/j.1538-7836.2009.03665.x 19874459

[50] Matsui T Titani K Mizuochi T. Structures of the asparagine-linked oligosaccharide chains of human von Willebrand factor, occurrence of blood group A, B, and H(?) structures. J Biol Chem. (1992) 267:8723–31. 1577715

[51] Sodetz JM Paulson JC McKee PA. Carbohydrate composition and identification of blood group A, B, and H oligosaccharide structures on human Factor VIII/von Willebrand factor. J Biol Chem. (1979) 254:10754–60. 315409

[52] Hironaka T Furukawa K Esmon PC Fournel MA Sawada S Kato M. Comparative study of the sugar chains of factor VIII purified from human plasma and from the culture media of recombinant baby hamster kidney cells. J Biol Chem. (1992) 267:8012–20. 1569060

[53] Matsui T Fujimura Y Nishida S Titani K Human plasma α_2_-macroglobulin and von Willebrand factor possess covalently linked ABO(H) blood group antigens in Subjects with corresponding ABO phenotype. Blood. (1993) 82:663–8. 7687165

